# Vγ2 x PD-L1, a Bispecific Antibody Targeting Both the Vγ2 TCR and PD-L1, Improves the Anti-Tumor Response of Vγ2Vδ2 T Cell

**DOI:** 10.3389/fimmu.2022.923969

**Published:** 2022-06-17

**Authors:** Rui Yang, Qing He, Hui Zhou, Cheng Gong, Xing Wang, Xingpan Song, Fang Luo, Yang Lei, Qian Ni, Zili Wang, Shasha Xu, Yan Xue, Man Zhang, Haimei Wen, Lijuan Fang, Liang Zeng, Yongxiang Yan, Jian Shi, Jing Zhang, Jizu Yi, Pengfei Zhou

**Affiliations:** Department of Early Discovery and Research, Wuhan YZY Biopharma Co., Ltd, Wuhan, China

**Keywords:** [Vγ2 x PD-L1], Vγ2Vδ2 T cell, PD-L1, adoptive transfer, immunotherapy

## Abstract

The potent cytotoxic property of Vγ2Vδ2 T cells makes them attractive for adoptive T cell transfer therapy. The transfusing of the expanded Vγ2Vδ2 T cells into cancer patients shows well-tolerated, but the clinical response rates are required to be improved, implying that there is still an unmet efficacy with low toxicity for this novel anti-tumor therapy. In this study, we test the anti-tumor efficacy of a Y-body-based bispecific antibody (bsAb) Vγ2 x PD-L1 that preferentially redirects Vγ2Vδ2 T cells to combat PD-L1 positive tumor cells. With nanomolar affinity levels to Vγ2Vδ2 T cells and PD-L1+ tumor cells, Vγ2 x PD-L1 bridges a Vγ2Vδ2 T cell with a SKOV3 tumor cell to form a cell-to-cell conjugation. In a PD-L1-dependent manner, the bsAb elicits effective activation (CD25+CD69+), IFNγ releasing, degranulation (CD107a+), and cytokine production (IFNγ+ and TNFα+) of expanded Vγ2Vδ2 T cells. The activations of the Vγ2Vδ2 T cells eliminate PD-L1-expressing human cancer cell lines, including H1975, SKOV3, A375, H1299, and H2228 cells, but not PD-L1 negative cells including HEK-293 (293) cells and healthy PBMCs. Finally, we show that combining Vγ2 x PD-L1 with adoptively transferring Vγ2Vδ2 T cells inhibits the growth of existing tumor xenografts and increases the number of Vγ2Vδ2 T cells into the tumor bed. Vγ2 x PD-L1 represents a promising reagent for increasing the efficacy of adoptively transferred Vγ2Vδ2 T cells in the treatment of PD-L1 positive malignant tumors.

## Introduction

Vγ2Vδ2 T cells, a unique fast-acting subset of innate γδ T cells found exclusively in primates (1), have been widely employed for adoptive cell immunotherapy in clinical studies for treating malignancies in past years (2). These cells have NK and cytotoxic T cell features, as well as potential and intrinsic rapid anti-tumor effector capabilities (3, 4), and appear to be a more promising candidate for allogeneic T cell therapy than αβ T cell-based CAR-T cells by participating in immune surveillance and killing a broad spectrum of cancer cells through a major histocompatibility complex (MHC)-independent activation mechanism (5). Recently, the adoptive transfer of Vγ2Vδ2 T cells to cancer patients has recently been shown to extend the survivals of late-stage liver cancers (23.1 vs 8.1 months) and lung cancers (19.1 vs 9.1 months) (6), and well tolerated as well (7). Yet, this therapy provided moderate clinical benefits with stable disease being the mostly outcome for patients who respond to this therapy (7).One of the reasons for this suboptimal effectiveness is the hostile tumor microenvironment that negatively regulates the anti-tumor functional characteristics of Vγ2Vδ2 T cells by the engagement between the programmed death-ligand 1 (PD-L1) expressed on the tumor cells and PD-1 expressed on the Vγ2Vδ2 T cells (8). Several groups proposed a combination approach of the Vγ2Vδ2 T cell-based adoptive immunotherapy with a PD-1 checkpoint blockade for the immunity against leukemia (9), follicular lymphoma (10), and prostate cancer (11). Likely, the anti-PD-L1 mAb enhances the cytotoxicity of Vγ2Vδ2 T cells against PD-L1^high^ cancer cells by adding ADCC activity (12).

After the success of targeting PD-1/PD-L1 axes, extensive efforts were directed to explore bsAb-based strategies to increase the anti-tumor activity of the adoptively transferred Vγ2Vδ2 T cells (13, 14). As a result, two representative series of Vγ2Vδ2 T cell-targeting bsAbs were constructed, one targeting to Vγ2-TCR and the other targeting to Vδ2-TCR. BsAb [(Her-2)_2_ × Vγ2] increased the cytotoxicity of Vγ2Vδ2 T cell against Her2-overexpressing pancreatic, ovarian and breast cancer cells showed by *in vitro* assay and in a PDAC grafted mouse model (15, 16). Similarly, Vγ2 x CD123 was created to treat acute myeloid leukemia (17). Lately, Vδ2 x EGFR elicits Vγ2Vδ2 T cell-mediated killing of colon cancer cell line SW480 both *in vitro* and *in vivo* (18), Vδ2 x CD1d for chronic lymphocytic leukemia (19), and Vδ2 x CD40 for b-cell malignancies (20). Moreover, these Vγ2Vδ2 T cell-specific targeting strategies were thought to overcome T cell over-activation induced by current CD3-targeting bsAbs, which could lead to cytokine storm syndrome, a severe side effect due to Treg stimulation. For example, the FDA-approved CD3 x CD19 bsAb, blinatumomab, could increase the numbers of Treg cells, which were correlated with non-responsiveness to blinatumomab in ALL patients (21) and further led to abnormal macrophage activation-dependent cytokine storm syndrome (22). Taken together, T cell engagers designed to activate Vγ2Vδ2 T cells exclusively might represent a feasible approach balanced between efficacy and safety.

Here, we describe the preclinical evaluation of Vγ2 x PD-L1. Our findings reveal that Vγ2 x PD-L1 activates selectively the fresh and expanded Vγ2Vδ2 T cells to kill tumor cells *in vitro*, enhances the migration of the transfused Vγ2Vδ2 T cells into tumor sites, and inhibits the growth of the existing tumors in nude mice. These data suggest that Vγ2 x PD-L1 plus adoptively transferred Vγ2Vδ2 T cells is potential to treat PD-L1 positive solid malignancies.

## Materials and Methods

### Generation of the Recombinant Antibodies

The bsAbs, including Vγ2 x PD-L1 and Vγ2 x Null, were generated similarly to Y111 described previously by Yang et al. (23). Briefly, the expression plasmids for Vγ2 x PD-L1 and Vγ2 x Null were synthesized and verified by sequencing in AuGCT Biotech (Wuhan, China). Then these expression vectors were transfected into cGMP banked CHO-S cells (Invitrogen, Carlsbad, USA) using the Fecto PRO Reagent (Ployplus, New York, USA) according to the manufacturer’s protocol, respectively. After a week, the cell culture supernatant was collected and serially purified by Sepharose Fast Flow protein A affinity chromatography column (GE, Milwaukee, USA), Fab Affinity KBP Agarose High Flow Resin (ACROBio systems, Newark, USA), and SP cation exchanged chromatography column (GE, Milwaukee, USA). Finally, the purified proteins were analyzed by SDS-PAGE and size-exclusion chromatograms. The Vγ2 x Null served as the control molecule for Vγ2 x PD-L1, with both molecules sharing the same backbone and Vγ2-targeting scFv part. Similarly, its two parental monoclonal antibodies (Vγ2 mAb (Clone 7A5) and PD-L1 mAb (23)) were produced.

### Tumor Cell Lines Culture

Tumor cell lines, including NCI-H1975 (human adenocarcinoma epithelial cell line, CRL-5908), SKOV3 (human ovarian adenocarcinoma cell line, HTB-77), A375 (human malignant melanoma cell line, CRL-1619), NCI-H1299 (human NSCLC metastatic cell line, CRL-5803), NCI-H2228 (human NSCLC adenocarcinoma cell line, CRL-5935), and nonmalignant kidney cell line HEK-293 were purchased from ATCC (Manassas, USA) and used as target cells. These cell lines were first transduced with firefly luciferase gene-containing pseudo-typed lentiviral particles purchased from GeneCopoeia (Shanghai, China), and the stable luciferase-expression cells were then selected under pressure of puromycin (Gibco, New York, USA). CHO-PD-L1 was generated from the parental CHO-K1 cell line (CCL-61, ATCC) through over-expressing human PD-L1. Tumor cells were cultured in RPMI 1640 (Biosharp, Hefei, China), DMEM or F-12K medium (purchased from Hyclone, New York, USA) supplemented with 10% FBS (Excell, Clearwater, USA) and penicillin/streptomycin (Gibco, New York, USA) and maintained in a humidified incubator with 5% CO_2_ at 37 °C. All cell lines in use were routinely tested for Mycoplasma infection using a commercial PCR kit (Vazyme, Nanjing, China), and new cultures were established monthly from frozen stocks as described previously (24).

### Expansion of Vγ2Vδ2 T Cells

The sampling protocols for human blood and *in vitro* experimental procedures were evaluated and approved by the institutional review boards for human subjects’ research and institutional biosafety committees at Hubei Province Food and Drug Safety Evaluation Center (Wuhan, China). All subjects are volunteer adults who signed on the informed consent.

Frozen or fresh human peripheral blood mononuclear cells (PBMCs) were obtained from LeiDeBio (Guangzhou, China) or Milestone (Shanghai, China). The *ex vivo* expansion protocol was described previously (23, 25). Briefly, PBMCs were cultured in RPMI 1640 medium (Gibco, New York, USA) supplemented with 10% FBS (Excell, Clearwater, USA), at 2×10^6^ cells/mL with the stimulation of 2.5 μM Zoledronic Acid (Sigma Aldrich, Darmstadt, Germany) and 1000 IU/mL IL2 (Sihuan Pharma, Beijing, China) for 10-14 days. The expanded Vγ2Vδ2 T cells were negatively enriched from the cultures by a TCR γ/δ + T Cell Isolation Kit (Miltenyi Biotech, Teterow, Germany). The purity and quality of the isolated cells were assessed by surface staining Vγ2/Vδ2 and CD86/CD69/HLA-DR as described previously ( (23)). Then, the purified Vγ2Vδ2 T cells were maintained in RPMI 1640 medium supplemented with 10% FBS overnight for rest before use. In this study, effector Vγ2Vδ2 T cells were expanded and purified from a total of eight healthy individuals for *in vitro* functional analysis and two healthy donors for *in vivo* anti-tumor evaluations.

### Binding Ability of Antibodies to Cells

A flow cytometry-based method was used to determine the affinities of Vγ2 x PD-L1 of its anti-Vγ2 arm to Vγ2Vδ2 T cells and its anti-PD-L1 arm to PD-L1 positive tumor cells. The sorted Vγ2Vδ2 T cells or tumor cells were incubated with serially diluted antibodies (Vγ2 x PD-L1, Vγ2 x Null, Vγ2 mAb, and PD-L1 mAb) for one hour at 4°C. After wash, the cells were stained for 30 minutes at room temperature with APC or PE-conjugated mouse-anti-human IgG Fc antibody (HP6017, Biolegend, San Diego, USA) diluted in 1:100. The cells were then resuspended in 200 μL FACS buffer (PBS with 2% FBS) and analyzed by a BD FACSelesta flow cytometer. For tumor cells, the cell-bound antibodies were quantified by the median fluorescence intensity (MFI) values, and the MFI were plotted against antibody concentrations to obtain the EC_50_. For Vγ2Vδ2 T cells, APC positive populations were used to determine the specific binding%.

The formation of an in-tans bridge between T cells and tumors cells was accessed by a flow cytometry method. Briefly, SKOV3 cells were stained with 50 nM CFSE, and the PBMC cultures (treated by Zol+IL2 for 10-14 days) were labeled by PKH26 according to the manufacturer’s protocol. Then, the CFSE-stained SKOV3 cells were co-cultured with PKH26-labelled PBMC cultures at a ratio of 1:1 with 1 ug/mL of Vγ2 x PD-L1 or Vγ2 x Null at an incubator for 0.5 hours. After washing, the cells were recorded on the FACSelesta (BD, San Jose, USA). The percentages of the CFSE^+^PKH26^+^ double-positive cells among the total cells have represented the ratios of cells engaged in cell-to-cell association.

### PD-L1 Blockade Reporter Assay

The assay was carried out following the manufacturer’s instructions (Promega, Cat#J1250). Briefly, PD-L1 aAPC/CHO-K1 cells were seeded at 4 × 10^4^ cells/well at 100 μL in white 96-well plates followed by a cultured overnight in an incubator at 37 °C with 5% CO2. The next day, the supernatant was discarded and the PD-L1 aAPC/CHO-K1 cells were incubated with serially diluted antibodies and PD-1 effector cells (5 × 10^4^/well) for 6 h. Then the relative luminescence units (RLU) of each well were determined using a Bio-Luc kit from Vazyme (Nanjing, China).

### PD-L1 Expression Scores Determination

Tumor cell lines were incubated with 40 μg/mL Vγ2 X PD-L1 (Target) or Vγ2 X Null (Null) for 1 hour at 4°C, then stained with APC-conjugated mouse-anti-human IgG Fc antibody (HP6017, Biolegend, San Diego, USA) for 30 minutes at room temperature. The APC positive populations and MFI of the APC channel were determined by flow cytometry. The expression scores were defined by [log_10_ (Target _APC positive populations_ - Null _APC positive populations_) + log_10_ (Target _APC MFI/_Null _APC MFI_)]/2.

### Evaluate T Cell Activation by Surface Staining and Intracellular Cytokine Staining

Flow cytometry was performed to evaluate T-cell activation as described in the other reports (26, 27). Expanded Vγ2Vδ2 T cells were enriched from PBMCs cultures (Zol+IL2 for 10-14 days), and cultured overnight. In parallel, 0.2 million H1975 or SKOV3 cells were plated in a 24-well-plate overnight. For activation assay, 0.2 million expanded and negatively enriched Vγ2Vδ2 T cells were added into either the tumor cell wells or empty wells with 1 μg/mL of Vγ2 X PD-L1 or Vγ2 X Null for 24 hours. Then, the cells were collected for staining FITC-anti-Vδ2 (B6, Biolegend, San Diego, USA), APC-anti-CD25 (M-A251, BD, San Jose, USA), and PE-anti-CD69 (FN50, BD, San Jose, USA) for 20 min at room temperature in dark. After wash, these cells were analyzed using flow cytometry. For intracellular cytokine staining, 0.2 million of the expanded and negatively enriched Vγ2Vδ2 T cells were added into the tumor cell wells or empty wells with 1 μg/mL of Vγ2 X PD-L1 or Vγ2 X Null plus a master mix containing BV510-anti-CD107a (H4A3, Biolegend) and BFA (Golgi Plug, BD, San Jose, USA) for 4 hours at 37 °C in 5% CO_2_. Then the cells were stained with Zombie Fixable Viability Kit (Biolegend), incubated with APC-anti-CD3 (SP34-2, BD, San Jose, USA), PE-anti-Vδ2 (B6, Biolegend, San Diego, USA) for 20 min at room temperature in dark. After incubation, cells were washed twice in FACS buffer and permeabilized for 20 min at 4°C (Cytofix/Cytoperm, BD, San Jose, USA). Then, cells were incubated with BV650-anti-IFNγ (4S.B3, Biolegend, San Diego, USA), BV421-anti-TNFα (Mab11, Biolegend, San Diego, USA) in Perm/Wash buffer for 30 min at room temperature in dark. These cells were washed twice with Perm/Wash buffer and collected by a BD FACSelesta flow cytometry. Flow data were analyzed by FlowJo (BD, San Jose, USA).

### Antibodies Mediated Cytotoxicity *In Vitro*

Two *in vitro* methods including luciferase-activity based assays and CFSE-PI staining-based assay were developed to access the killing ability of Vγ2Vδ2 T cells mediated by antibodies.

Luciferase-activity based assays: 2 x 10^4^ firefly luciferase-expressing tumor cells (Target: T) were co-incubated with expanded Vγ2Vδ2 T cells (Effector: E) at an E:T ratio of 0.5:1 (or other indicated E: T ratios), or fresh enriched γδ T cells (Effector) at an E:T ratio of 5:1, in the presence of a serial of diluted antibodies for 12 hours in a white 96-well-flat bottom plate. A Bio-Luc kit from Vazyme (Nanjing, China) was used to measure luciferase activity. Then the “Specific lysis” was calculated as follows: % Specific lysis = [1 – (RLU _Ab-treated wells_)/(RLU _Target-only wells_)] × 100.

CFSE-PI staining-based assay: Unrelated healthy PBMCs were stained with CFSE according to the manufacturer’s protocol. Then these cells were co-cultured with Vγ2Vδ2 T cells at a 1:1 E: T ratio in the presence of various doses of indicated antibodies for 12 hours. Then 1μg/mL of PI (Sigma) was added to the wells. The percentages of CFSE^+^PI^+^ cells among the total of target cells (CFSE^+^) were defined as “Specific Cytotoxicity%” values.

### Measuring Vγ2Vδ2 T Cell Releasing IFNγ

The supernatant was collected from T cell and tumor cell co-culture wells and stored at -80°C until measurement. Human IFNγ were quantified with the ELISA kits from Proteintech (KE00063, Wuhan, China).

### Mouse Tumor Model

Female nude mice were obtained from the VITALSTAR (Beijing, China) at age of 6-8 weeks and were used in this study under a protocol approved by the Animal Care and Use Committee from Hubei Province Food and Drug Safety Evaluation Center (#202110191).

Firstly, 5 million SKOV3 cells were subcutaneously inoculated into the right dorsal flank of nude mice on Day 0. After one week, tumor volumes had reached around 200 mm^3^, these mice were randomly divided into three groups receiving PBS, 2 million purified Vγ2Vδ2 T cells i.v. through lateral tail vein plus 8 mg/kg Vγ2 X Null i.p. or 8 mg/kg Vγ2 X PD-L1 *i.p*. on Days 7,11,14, and 18 (Q2W, two weeks, four times). After treatment, tumor volumes and mice body weights were measured three times a week. The tumor volume was calculated using the formula: Tumor Volume (mm^3^) = (a x b^2^)/2, where a is the longitudinal length and b is the transverse width. On day 34, these mice were sacrificed and tumor xenografts were excised for tumor weighting and IHC staining.

### IHC Analysis

The tumor tissues were cut into small pieces embedded in 4% paraformaldehyde for fixation. Then these tumor pieces were sectioned and examined by IHC staining using a rabbit-anti-human CD3 antibody (Clone SP7). Tissue sections were then counter-stained with hematoxylin. Positive cells were counted in five randomly selected microscopic fields (magnification 20X) and supplied for further quantification analysis.

### Statistical Analysis

Statistical analyses were performed with GraphPad Prism 6.0 (La Jolla, USA). Before performing nonlinear regression analysis for *in vitro* assays (cell binding and killing), the antibody concentrations (on the x-axis) were transformed in a log scale. Then, the “log (agonist) vs. response- Variable slope (four parameters)” method was applied to calculate EC_50_. P values were assessed by one-way or two-way ANOVA, followed by Dunnett test or Tukey multiple comparisons as appropriate. P values <0.05 were considered to be significant. P values were reported in **Supplementary Table 1**

## Results

### Design, Generation, and Characterization of Vγ2 x PD-L1

**Figure 1:**
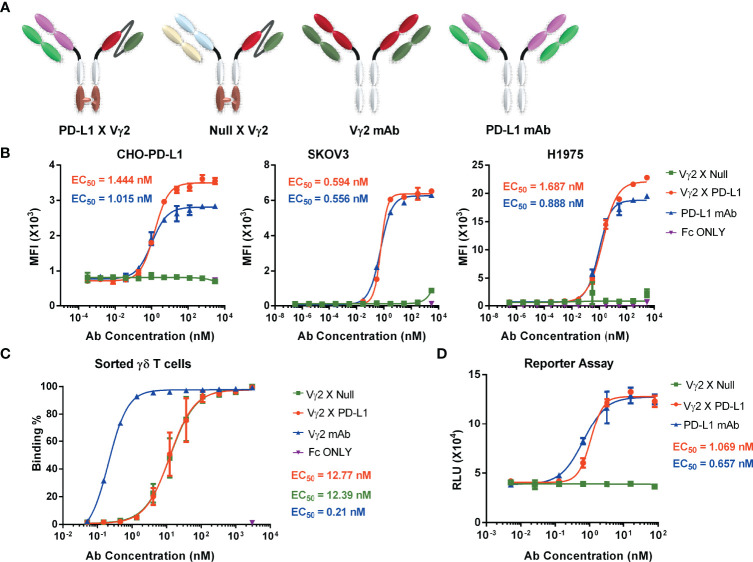
PD-L1 x Vγ2 interacts Vγ2Vδ2 T cells and PD-L1 expressing tumor cells, and blocks the PD1/PD-L1 interaction. **(A)** Structural diagrams of bispecific antibodies, including PD-L1 x Vγ2, Null x Vγ2, Vγ2 mAb, and PD-L1 mAb. A “Knob-into-hole” in Fc region was introduced into the bsAbs (6, 28). Besides, these four antibodies contained a modified silent Fc fragment to abolish Fc-mediated effector functions (6, 28). Please noted that Vγ2 mAb and PD-L1 mAb, targeting Vγ2-TCR and PD-L1, respectively, are parental monoclonal antibodies; Vγ2 x Null, targeting Vγ2 and fluorescein (29), and Vγ2 x PD-L1, targeting Vγ2-TCR and PD-L1. The purity of these prepared antibodies was shown in **Supplementary Figure 1**. **(B)** Binding affinity to PD-L1 positive cell lines. CHO-PD-L1, SKOV3, and H1975 cells were incubated with serially diluted antibodies, followed by PE-labelled mouse-anti-human Fc secondary antibody. Mean fluorescence intensity (MFI) of the PE channel of each sample was measured to determine specific binding ability (EC50). These three cell lines were PD-L1 positive shown in **Supplementary Figure 2A**. **(C)** Antibody binding affinity to Vγ2Vδ2 T cells. Vγ2Vδ2 T cells were negatively enriched from PBMC cultures treated by Zol+IL2 for 14 days. Then, cells were incubated with serial dilutions of indicated antibodies, followed by APC-conjugated mouse-anti-human Fc secondary antibody. APC positive populations were measured to demonstrate specific binding (EC50). The representative flow cytometry plots related **(B, C)** were shown in **Figure 2**. **(D)** The ability of PD-L1 x Vγ2 to block PD1/PD-L1 signaling (EC50) was similar to that of the parental PD-L1 mAb using a cell-based reporter assay. Data were presented as Mean ± SD from n = 3 independent experiments **(B, D)**, pooled from n=1 biological replicate for Vγ2 mAb, n=6 biological replicates for Vγ2 x Null and Vγ2 x PD-L1 **(C)**. Reported EC_50_ values were calculated from non-linear best fits **(B–D)**.

**Figure 2:**
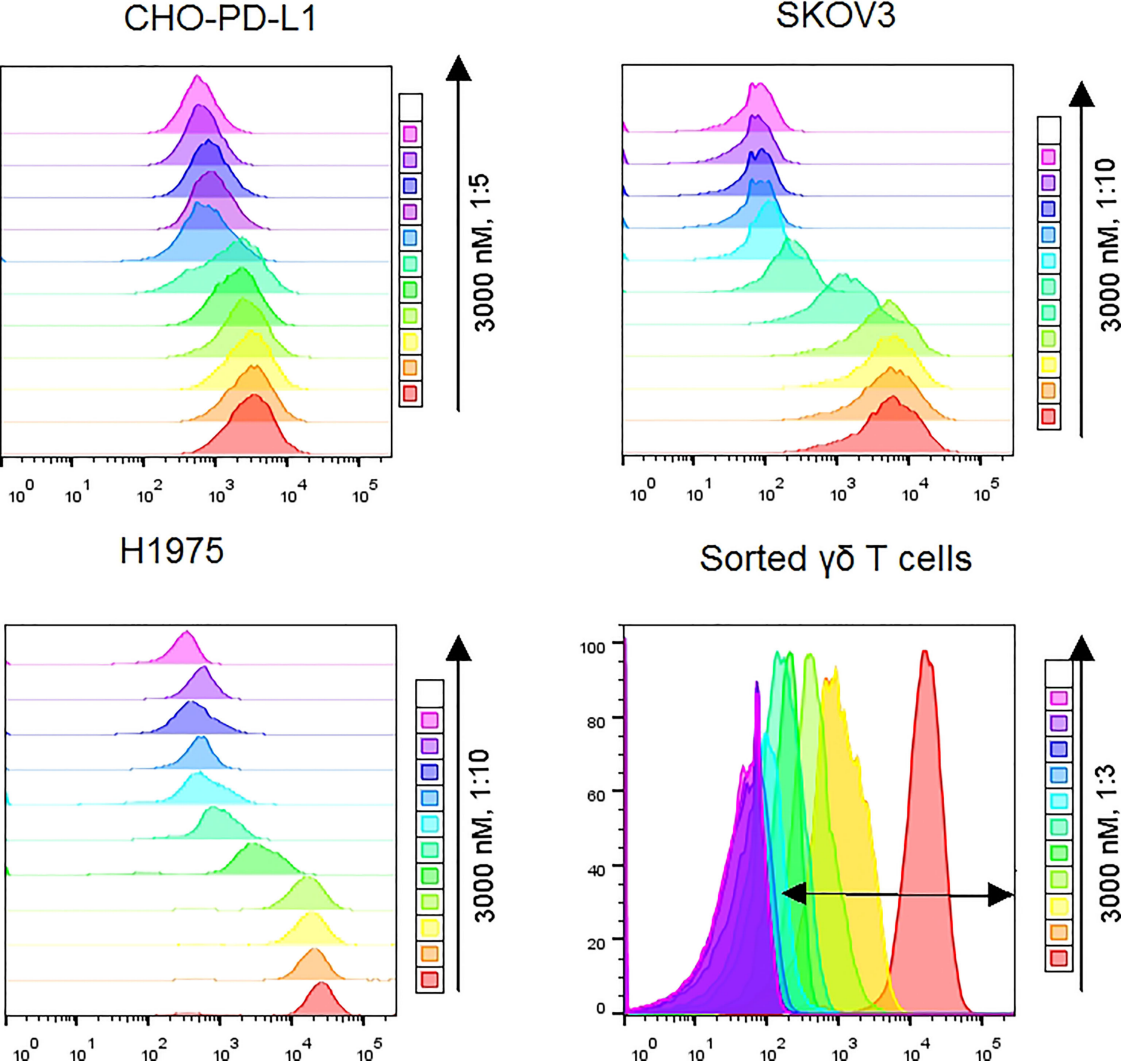
Representative flow cytometry plots showed MFI differences along with doses of Vγ2 x PD-L1 for CHO-PD-L1, SKOV3, and H1975 cells. For sorted γδ T cells, the double-headed arrow indicated the APC positive population.

We initially designed and constructed four recombinant antibodies, i.e. Vγ2 x PD-L1, Vγ2 x Null, PD-L1 mAb and Vγ2 mAb to test their activities. The structural properties of these generated antibodies were summarized in **Figure 1A**. Firstly, the molecular weights of these recombinant proteins were confirmed through SDS-PAGE under both reducing and non-reducing conditions (**Supplementary Figure 1A**). Then, the SEC results indicated that the purities of the prepared antibodies were more than 95% (**Supplementary Figure 1B**). Next, we used three PD-L1 expression cell lines (CHO-PD-L1, SKOV3, and H1975) to compare antibody binding ability to the cells between Vγ2 x PD-L1 and PD-L1 mAb. The mean EC_50_ values for Vγ2 x PD-L1 binding to CHO-PD-L1, SKOV3, and H1975 were 1.444 nM, 0.594 nM, and 1.687 nM, respectively (**Figure 1B**, **Figure 2**). Both Vγ2 x PD-L1 bsAb and PD-L1 mAb had a similar affinity to the cellular surface PD-L1 (**Figure 1B**), due to these two antibodies having the same variable regions for PD-L1 binding (23). Furthermore, we determined the PD-L1 expression scores for a series of target tumor cells using Vγ2 x PD-L1 bsAb, which confirmed that Vγ2 x PD-L1 exhibited potent affinity toward tumor cells with variable PD-L1 expression levels (**Supplementary Figure 2**). In addition, the binding affinity to the expanded Vγ2Vδ2 T cells of Vγ2 x PD-L1 was about 60-folds weaker than that of the parental Vγ2 mAb, as the mean EC_50_ values for Vγ2 x PD-L1 and Vγ2 mAb were 12.39 nM and 0.21 nM, respectively (**Figures 1C**, **2**). Moreover, Vγ2 x PD-L1 retained the blocking ability as PD-L1 mAb, which was demonstrated in the PD1/PD-L1 cell-based reporter assay (**Figure 1D**). In summary, Vγ2 x PD-L1 bound with nanomolar affinity to the sorted and expanded Vγ2Vδ2 T cells and PD-L1 expressing tumor cells.

### Vγ2 x PD-L1 Efficiently Bridges Vγ2Vδ2 T Cells to PD-L1 Positive Tumor Cells

Subsequently, we checked whether the Vγ2 x PD-L1 prompted the formation of the biphasic cell-to-cell conjugates between Vγ2Vδ2 T cells and PD-L1 expressing tumor cells. For this purpose, Vγ2Vδ2 T cells stained with CFSE were co-cultured with PKH26-labelled SKOV3 cells for 30 minutes at 37°C with Vγ2 x PD-L1 or Vγ2 x Null, then the percentages of double-positive cells among total cells were measured to represent the bridging ability. In the presence of Vγ2 x Null at 1 μg/mL, the double-positive cell population (Q2) was 2.21%, while this population was increased up to 20.1% by Vγ2 x PD-L1 (**Supplementary Figure 3**). In contrast, Vγ2 x PD-L1 failed to prompt the co-binding of Vγ2Vδ2 T cells and HEK-293 cells. (**Supplementary Figure 3**)

### Vγ2 x PD-L1 Selectively Activates Vγ2Vδ2 T Cells Exposed to PD-L1 Expressing Tumor Cell Lines

**Figure 3:**
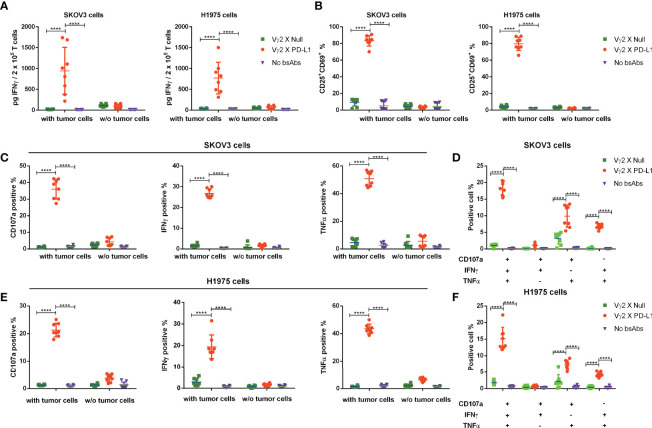
Vγ2 x PD-L1 revoked specifically activation, of the expanded Vγ2Vδ2 T cells in the presence of PD-L1^+^ tumor cell lines. **(A)** Vγ2 x PD-L1 increased significantly the IFNγ secretion and **(B)** prompted activation of the expanded Vγ2Vδ2 T cells in a PD-L1-dependent fashion. Vγ2Vδ2 T cells were co-cultured with indicated tumor cell lines (SKOV3 or H1975) in the absence or presence of Vγ2 x PD-L1 or Vγ2 x Null (1 μg/mL of each, about 8 nM) at a ratio of 1:1 for 24 hours. Then the supernatant was harvested for measuring the concentration of IFNγ by ELISA **(A)**, and cells were collected for staining CD25+CD69+ double-positive populations **(B)**. **(C–F)** Vγ2 x PD-L1 activated specifically Vγ2Vδ2 T cells to produce IFNγ and TNFα, and degranulate in the presence of PD-L1^+^ tumor cell lines. Vγ2Vδ2 T cells were stimulated by Vγ2 x PD-L1 or Vγ2 x Null (1 μg/mLof each) in the presence/absence of H1975 **(C, D)** or SKOV3 **(E, F)** cells in a 1:1 ratio for 4 hours. The percentages of T cells positive for CD107a, TNFα, and IFNγ measured by ICS were represented in **(C, E)** and the percentages of multi-functional effector subsets of Vγ2Vδ2 T cells were shown in **(D, F)** Data were presented as Mean ± SD pooled from n=8 biological replicates of three independent experiments. ****p< 0.0001 (Two-way ANOVA, Tukey’s multiple comparisons test for **(A, B, C, E)** Dunnett’s multiple comparisons test for **(D, F)**.

**Figure 4:**
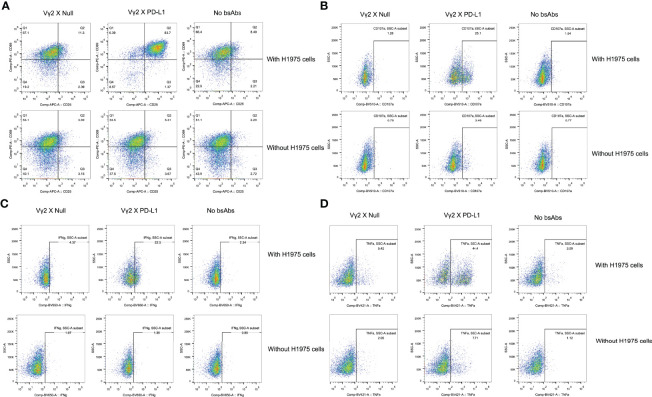
Representative flow cytometry plots. The plots were showed for the activation **(A)**, TNFa and IFNg production **(B, C)**, and CD107a upregulation **(D)** of Vg2Vd2 T cells as under the indicated conditions.

Next, we investigated whether the activation of Vγ2Vδ2 T cells mediated by Vγ2 x PD-L1 was dependent on the presence of PD-L1^+^ tumor cells. Vγ2Vδ2 T cells were co-cultured with H1975 and SKOV3 cells, the two cell lines that expressed high levels of PD-L1 (**Supplementary Figure 2A**). Vγ2Vδ2 T cells secreted little amount of IFNγ and did not exhibit activation phenotype (measured by CD25^+^CD69^+^) in response to the bsAbs treatment alone (**Figures 3A, B**, **Figure 4**). Of note, in the presence of H1975 and SKOV3 cells, Vγ2 x PD-L1, but not Vγ2 x Null, triggered significantly the release of IFNγ and active phenotype of Vγ2Vδ2 T cells (**Figures 3A, B**, **4**). Accordingly, Vγ2 x PD-L1 further enhanced significantly both the IFNγ and TNFα productions and degranulation levels of Vγ2Vδ2 T cells only in the presence of PD-L1 positive SKOV3 and H1975 cells (**Figures 3C-F**, **4**). Moreover, these Vγ2Vδ2 T cells activated jointly by Vγ2 x PD-L1 and PD-L1 tumor cells displayed multifunctional effector phenotypes, which co-expressed IFNγ, TNFα, and CD107a (**Figures 3D, F**, **Figure 4**). In contrast, Vγ2 x Null did not exert agonistic effects on Vγ2Vδ2 T cells even when co-cultured with PD-L1 expressing target cells in the above conditions (**Figures 3C-F**, **4**). Together, these data demonstrated that Vγ2 x PD-L1 revoked robust effector functions of Vγ2Vδ2 T cells, including activation, degranulation, and cytokines secretion, in dependent on the engagement of target tumor cells.

### Vγ2 x PD-L1 Induces PD-L1^+^ Tumor Cell Lysis at a Lower E: T Ratio

**Figure 5:**
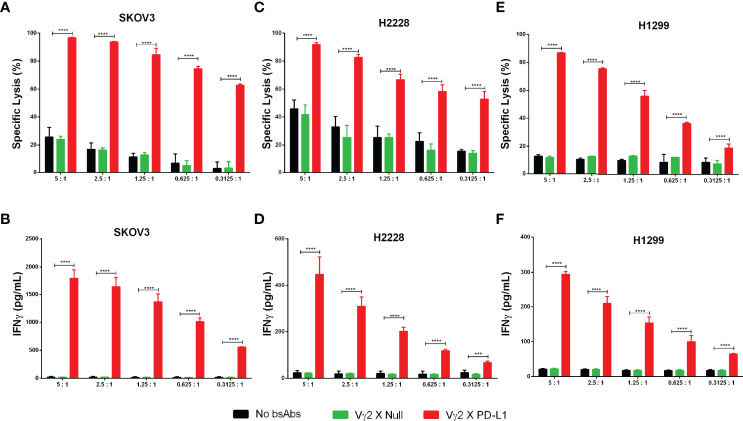
Vγ2 X PD-L1 prompts significantly Vγ2Vδ2 T cell-mediated PD-L1+ tumor cell killing through releasing IFNγ. **(A–F)** Vγ2Vδ2 T cells enriched negatively from Zol+IL2 cultures were co-cultured with tumor targets (luciferase-expressing SKOV3, H2228, and H1299 cells) for 12 hours in the presence of 1 ug/mL (8 nM) Vγ2 X PD-L1 or Vγ2 X Null with serial E:T ratios, ranging from 5:1 to 0.3125:1. The tumor cell killing was measured by recording the RLU of each treated well **(A, C, E)**, and the releasing amounts of IFNγ were determined by ELISA **(B, D, F)**. Data were presented as Mean ± SD pooled from n=4 biological replicates of two independent experiments. ****p< 0.0001 (Two-way ANOVA, Dunnett’s multiple comparisons test). ***p<0.001.

Then, we assessed whether Vγ2 x PD-L1 could lysis of tumor cells with variable PD-L1 expressing levels. To this end, Vγ2Vδ2 T cells were co-cultured with SKOV3, H2228, and H1299 cell lines in E:T ratios ranging from 5:1 to 0.3125:1 for 12 hours. We selected SKOV3, H2228 for this test as these two cell lines expressed PD-L1 at high or low levels as determined using Vγ2 x PD-L1 staining (**Supplementary Figure 2B**). Vγ2Vδ2 T cells alone showed E: T ratio-dependent cytotoxicity for SKOV3 and H2228 (**Figures 5A, B**). The addition of Vγ2 x PD-L1, but not Vγ2 x Null, significantly enhanced tumor cell death even at the lowest E: T ratio (0.3125:1) for the both cell lines (**Figures 5A, B**). Furthermore, the larger amount of IFNγ was only detected in the Vγ2 x PD-L1 treated cultures, demonstrating that Vγ2 x PD-L1 elicited PD-L1-specific IFNγ production from Vγ2Vδ2 T cells (**Figures 5C, D**). We then evaluated whether Vγ2 x PD-L1 could enhance cytotoxicity towards tumor cells that were resistant and refractory to Vγ2Vδ2 T cells’ killing. Indeed, Vγ2Vδ2 T cell alone lysed less than 20% of H1299 cells even at a 5:1 ratio (**Figure 5E**). However, Vγ2 x PD-L1 strongly increased the lysis of H1299 with the increased IFNγ production by Vγ2Vδ2 T cells (**Figures 5E, F**). Importantly, Vγ2 x PD-L1 induced efficient tumor cell lysis, and IFNγ secretion was observed at an E: T ratio as low as 0.3125:1 for these three cell lines (**Figure 5**).

### Vγ2 x PD-L1 Potency in Killing PD-L1 Positive Tumor Cell Lines Is Mediated by Both Fresh and Expanded Vγ2Vδ2 T Cell

**Figure 6:**
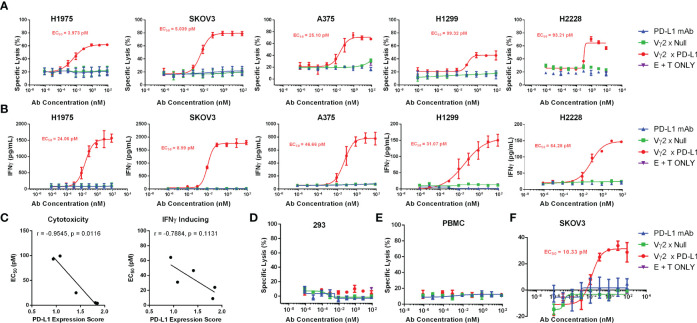
Vγ2 x PD-L1 redirects Vγ2Vδ2 T cells to kill efficiently various PD-L1 positive cancer cell lines *in vitro*, but spared this effect on PD-L1 negative expressing HEK-293 cells or unrelated healthy PBMCs. **(A)** Expanded Vγ2Vδ2 T cells derived from healthy donors’ PBMCs (n=3) were incubated with various luciferase-expressing tumor cell lines at a 0.5:1 ratio under the stimulation of serial concentrations of antibodies, including Vγ2 x PD-L1, Vγ2 x Null or PD-L1 mAb, for 12 hours. **(B)** Increased IFNγ secretion in the above co-cultures. **(C)** The cytotoxicity and IFNγ induction of Vγ2Vδ2 T cells revoked by Vγ2 x PD-L1 correlated with the PD-L1 expression score. The spearman’s r and two-tailed p values were calculated by GraphPad Prism 6. **(D, E)** The expanded Vγ2Vδ2 T cells derived from healthy donors’ PBMCs (n=3) were incubated with CFSE-labelled PD-L1^neg^ HEK-293 **(D)** or allogeneic PBMCs **(E)** at 1:1 ratio as indicated in **(A)**. A CFSE/PI staining-based flow cytometry method to determine the killed target cell percentages. **(F)** Fresh Vγ2Vδ2 T cells enriched from healthy donors (n=2) were incubated with SKOV3-Luc at 5:1 ratio under the stimulation of serial concentrations of antibodies, including Vγ2 x PD-L1, Vγ2 x Null or PD-L1 mAb, for 12 hours.

To confirm whether Vγ2 x PD-L1 could redirect Vγ2Vδ2 T cells to kill a broad spectrum of tumor cells, we took 5 different human solid tumor cell lines expressing PD-L1 for the test. For these PD-L1 expressing tumor cells, Vγ2Vδ2 T cells alone did not exert an appreciable killing effect, nor did the PD-L1 mAb (**Figure 6A**). However, a dose-dependent effective killing mediated by Vγ2Vδ2 T cells was observed with the addition of Vγ2 x PD-L1 irrespective of tumor cells’ origin, but not for Vγ2 x Null (**Figure 6A**). As expected, Vγ2Vδ2 T cells exhibited a dose-dependent IFNγ secretion treated with Vγ2 x PD-L1, compared with no such effect with control Abs (**Figure 6B**). We further observed that the Vγ2 x PD-L1-induced Vγ2Vδ2 T cells’ cytotoxicity (killing EC_50_) towards tumor cells was correlated significantly with these tumor cells’ PD-L1 expression scores, while the release IFNγ EC_50_ showed a negative trend with the PD-L1 expression scores (**Figure 6C**). Moreover, the viability of PD-L1^neg^ HEK-293 cells remained unaffected in all tested concentrations in the presence of Vγ2 x PD-L1 (**Figure 6D**). In addition, allogeneic PBMCs were used as target cells to check if the killing activity of Vγ2 x PD-L1 was specific to tumor cells. The Vγ2Vδ2 T cell-mediated killing percentages of allogeneic PBMCs were low even in the presence of Vγ2 x PD-L1, indicating the Vγ2 x PD-L1 activated Vγ2Vδ2 T cells’ killing activity was indeed restricted to tumor cells (**Figure 6E**). Moreover, fresh Vγ2Vδ2 T cells enriched from healthy donors also exerted concentration-dependent killing of SKOV3 cells mediated by Vγ2 x PD-L1, but not by Vγ2 x Null or PD-L1 mAb (**Figure 6F**). Taken together, these results demonstrated that Vγ2 x PD-L1 could redirect Vγ2Vδ2 T cells to kill PD-L1+ tumor cell lines with IFNγ secretion, but to leave PD-L1 negative tumor cells and healthy cells un-attacked.

### Vγ2 x PD-L1 Enhances the Efficacy of Adoptively Transferred Vγ2Vδ2 T Cells *In Vivo*

**Figure 7:**
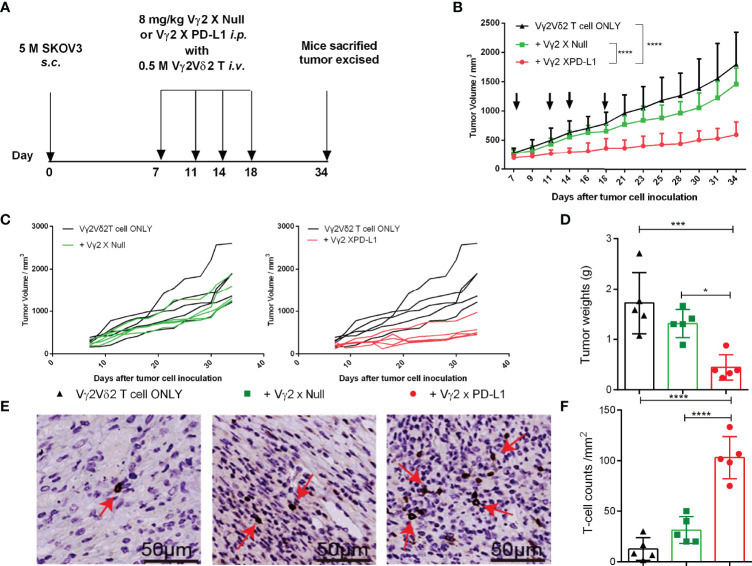
Vγ2 X PD-L1 prompted the survival of inoculated Vγ2Vδ2 T cells in nude mice. **(A)** Experimental schema of evaluating the anti-tumor therapeutic efficacy of Vγ2 X PD-L1. Nude mice were *s.c.* inoculated with 5 million SKOV3 cells on Day 0. After 15 days, mice were treated with *i.v.* Vγ2Vδ2 T cells plus 8 mg/kg Vγ2 X Null or Vγ2 X PD-L1. These treatments were repeated twice a week (Q2W) for 3 weeks. Mice treated PBS only were used as control. **(B, C)** Pooled or individual tumor growth curves. The black arrows indicated the treatment time point. Data are mean ± SD with 5 mice per group, ****p < 0.0001 (Two-way ANOVA, Dunnett’s test), which was determined based on the tumor volumes at the end of the study. **(D)** Tumor weights at the end of the study. Data were mean ± SD with 5 mice per group, ***p< 0.001, *p<0.05 (ANOVA, Dunnett’s test). **(E, F)** Infiltrated and accumulated T-cell counts at the tumor site. Representative IHC figures for the treated group **(E)** and pooled T cell counts **(F)** were presented as mean ± SD, ****p<0.0001, (ANOVA, Dunnett’s test). Data shown was one of two independent experiments.

We further studied the effect of Vγ2 x PD-L1 on the outgrowth of established PD-L1 expressing tumors. SKOV3 cells were injected into nude mice, and the tumor cells were allowed to grow out and engraft for one week before the mice received twice-weekly *i.v.* injections with human Vγ2Vδ2 T cells, followed by twice-weekly *i.p.* injections with either 8 mg/kg Vγ2 x PD-L1 or Vγ2 x Null, or PBS. The mice were sacrificed at the time of severe disease symptoms (**Figure 7A**). The Vγ2Vδ2 T cells alone, or Vγ2Vδ2 T cells plus Vγ2 x Null did not control the tumor growth (**Figures 7B, C**). In contrast, the combo treatment with Vγ2 x PD-L1 and Vγ2Vδ2 T cells significantly delayed the tumor growth, with lower tumor weights at the end of the study (**Figures 7B-D**) than those of the control groups. After 16 days of treatment, Vγ2Vδ2 T cell counts were significantly higher in the Vγ2 x PD-L1+Vγ2Vδ2 T cells group, compared with the Vγ2 x Null+ Vγ2Vδ2 T cells group or Vγ2Vδ2 T cells group (**Figures 7E, F**).

## Discussion

The clinical investigations of PD-1/PD-L1 inhibitors have resulted in a paradigm shift in the treatment of advanced cancer patients, as well as longer overall survival time (30). However, due to the limited efficacy (only 20 to 30% objected response) and resistance to PD-1/PD-L1, there is still an unmet medical need for exploring novel agents to improve PD-L1 targeting therapeutic effectiveness (31). The inadequate infiltration of T lymphocytes into the cold tumor is one of the reasons for this therapeutic resistance (32). Several clinical studies showed that transferred Vγ2Vδ2 T cells migrated into the tumor bed, leading to encouraging clinical responses and tumor reduction in treated patients (33). Here, the bispecific antibody and Vγ2Vδ2 T cells transfer combination approach provided a potential strategy to circumvent the PD-L1 blockade therapy limitations. The approach for targeting potent cytotoxicity Vγ2Vδ2 T cells by constructing Vγ2 x PD-L1 on the Y-body platform, based-on which two novel candidate medications are currently on clinical trials, noted as M701 (NCT04501744) and M802 (NCT04501770) (34). Vγ2 x PD-L1 preserved high affinity to PD-L1 as well as the PD1/PD-L1 blocking activity. However, consistent with other reports, the PD-1/PD-L1 blocking activity did not contribute to the killing ability of Vγ2Vδ2 T cells (12), possibly because the PD-L1 mAb used in our study contained silent Fc without ADCC capability. Vγ2 x PD-L1 had a slower affinity for the Vγ2 TCR than Vγ2 mAb, which was desired for clinical use to prevent cytokine release storm (35). Additionally, Vγ2 TCR-targeting Y-body platform allowed for the simple replacement of the PD-L1 Fab to create a sequence of Vγ2 x TAAs, which enabled Vγ2Vδ2 T cells to target a broader spectrum of tumor types and helping a larger population of cancer patients.

*In vitro*, Vγ2 x PD-L1-activated Vγ2Vδ2 T cells were able to selectively kill tumor cells selectively without killing PD-L1 negative non-malignant cells or normal cells. In fact, the activation, degranulation, and subsequent tumor cell killing mediated by Vγ2 x PD-L1 were all dependent on simultaneous binding to Vγ2Vδ2 T cell and PD-L1 expressing tumor cells, demonstrating the safety of our strategy in comparison to PD-L1 chimeric antigen receptor NK cells (36). In line with these *in vitro* observations, Vγ2 x PD-L1 was found to improve Vγ2Vδ2 T cell mediated tumor growth inhibition *in vivo*. Mechanically, Vγ2 x PD-L1 generated a greater Vγ2Vδ2 T cell infiltration.

Meanwhile, there are several limitations in this study. First, because Vγ2Vδ2 T cells are species specific, we employed an immunodeficiency mouse model to investigate the efficacy of Vγ2 x PD-L1 plus Vγ2Vδ2 T cells, without examining whether this combination therapy could change or reshape the suppressive tumor microenvironment, or the *in vivo* toxicity of combo usage. Second, this combo treatment was not fully curative because tumor volumes did not reach to near zero by the end of treatment. As a small amount of Vγ2Vδ2 T cells and a fixed bsAb dose were used in the current treatment protocol, we intended to improve the present therapy approach involving a modest number of Vγ2Vδ2 T cells and bsAb dosage. Third, we were unable to determine the TCR sequence of tumor bed infiltrating Vγ2Vδ2 T cells, which would provide valuable information for further TCR-T design.

In conclusion, we developed a novel and potential therapeutic T cell engager bispecific antibody Vγ2 x PD-L1, which caused Vγ2Vδ2 T cells to destroy PD-L1 expressing tumor cells efficiently and selectively. Vγ2 x PD-L1 offers promising therapy options for solid tumors, including ovarian cancer (28, 37, 38), melanoma (38, 39), and non-small cell lung cancer (NSCLC) (38). The infiltrating Vγ2Vδ2 T cells in tumor acted as protective anti-tumor effector population and were linked with positive outcomes. As PD-L1 is a clinically well-established tumor target, its widespread expression pattern suggested that our combination approach might be beneficial for the PD-L1 positive cancer patients who had refractory or relapsed for PD-L1 inhibitor treatment.

## Data Availability Statement

The raw data supporting the conclusions of this article will be made available by the authors, without undue reservation.

## Ethics Statement

The studies involving human participants were reviewed and approved by the institutional review boards for human subjects’ research and institutional biosafety committees at Hubei Province Food and Drug Safety Evaluation Center (Wuhan, China). The patients/participants provided their written informed consent to participate in this study. The animal study was reviewed and approved by the Animal Care and Use Committee from Hubei Province Food and Drug Safety Evaluation Center (#202110191).

## Author Contributions

RY, JY, and PZ conceived the ideas and designed the project. JZ, JS, YY, LF and LZ supervised the project. RY, YX, MZ, HW, HZ, CG, XW, FL, XS, ZW, SX, YL, and QN performed the experiments. RY, JY and PZ analyzed the data and jointly wrote the manuscript. All authors read and approved the manuscript. All authors contributed to the article and approved the submitted version.

## Funding

This work was supported partly by National Natural Science Foundation of China (81901607), China Postdoctoral Science Foundation (2021M692495), and the 3551 Optics Valley Young Talent Schema of Wuhan East Lake High-tech Development Zone.

## Conflict of Interest

The authors are employees of Wuhan YZY Biopharma Co., Ltd that develops and commercializes antibody therapeutics including bispecific antibodies.

## Publisher’s Note

All claims expressed in this article are solely those of the authors and do not necessarily represent those of their affiliated organizations, or those of the publisher, the editors and the reviewers. Any product that may be evaluated in this article, or claim that may be made by its manufacturer, is not guaranteed or endorsed by the publisher.

## Supplementary Material

Supplementary Figure 1 Biochemical analysis of generated Vγ2Vδ2 T cell-targeting bsAbs. **(A)** SDS-PAGE analysis of purified antibodies under non-reducing (left) and reducing (right) conditions. Molecular weight (MW) was indicated in kDa for protein marker. There were 3 and 2 bands for bsAb and mAb, respectively, under reducing conditions as expected. **(B)** Size exclusion chromatograms of test antibodies (upper, Vγ2 X PD-L1, bottom, Vγ2 X Null). The antibodies were purified by Protein-A and ion-exchange chromatography. The purity of prepared bsAb was more than 95%.

Supplementary Figure 2 PD-L1 expression scores of various tumor cell lines tested in this study. (A) Representative histogram of PD-L1 expression on CHO-PD-L1, H1975, and SKOV3 cells. Vγ2 X PD-L1 (blizzard blue shade), Vγ2 X Null (pink shade) of two experiments. (B) PD-L1 expression scores of firefly luciferase-transduced tumor cells. The CHO-PD-L1 cells were used as positive control, and HEK-293-Luc cells were displayed null expression of PD-L1 serving as negative control. Various tumor cell lines were incubated with 40 μg/mL Vγ2 X PD-L1 (Target) or Vγ2 X Null (Null) for 1 hour at 4 degree, then stained with APC-hFc for 30 minutes at room temperature. The APC positive populations and MFI of APC channel were determined by flow cytometry. The expression scores were calculated by [log_10_ (Target _APC positive populations_ - Null _APC positive populations_) + log_10_ (Target _APC MFI/_Null _APC MFI_)]/2. Data were derived from one representative experiment of three independent experiments.

Supplementary Figure 3 Vγ2 x PD-L1 recruited Vγ2Vδ2 T cells to form cell-to-cell conjugates with SKOV3 cells, but not with 293T cells. 0.1 M CFSE-labelled Vγ2Vδ2 T cells were incubated with 0.1 M PKH26-stained SKOV3 cells in the presence of 1 μg/mL (8 nM) Vγ2 x Null (Left) or Vγ2 x PD-L1 (Right) for 30 minutes, then the percentages of CFSE and PKH26 double positive cells (Q2) were depicted as cell-to-cell conjugates. Representative flow cytometric dot plots from three independent experiments (for SKOV3) and three wells (for HEK-293) were shown. Please noted that the upper panel used Vγ2Vδ2 T cells negatively enriched from fresh PBMC cultures treated with Zol+IL2 for 14 days, the bottom panel used Vγ2Vδ2 T cells enriched from cryopreserved PBMC cultures treated with Zol+IL2 for 14 days; and Vγ2Vδ2 T cells were expanded from different donors for SKOV3 and HEK-293.

Supplementary Table 1 Summary p values for **Figures 2**, **3**, **5**.
