# Supplementary figures and images for: Vγ2 x PD-L1, a Bispecific Antibody Targeting Both the Vγ2 TCR and PD-L1, Improves the Anti-Tumor Response of Vγ2Vδ2 T Cell
Source: Front Immunol. 2022 Jun 17;13:923969. doi: 10.3389/fimmu.2022.923969 (PMC9247338; doi:10.3389/fimmu.2022.923969)

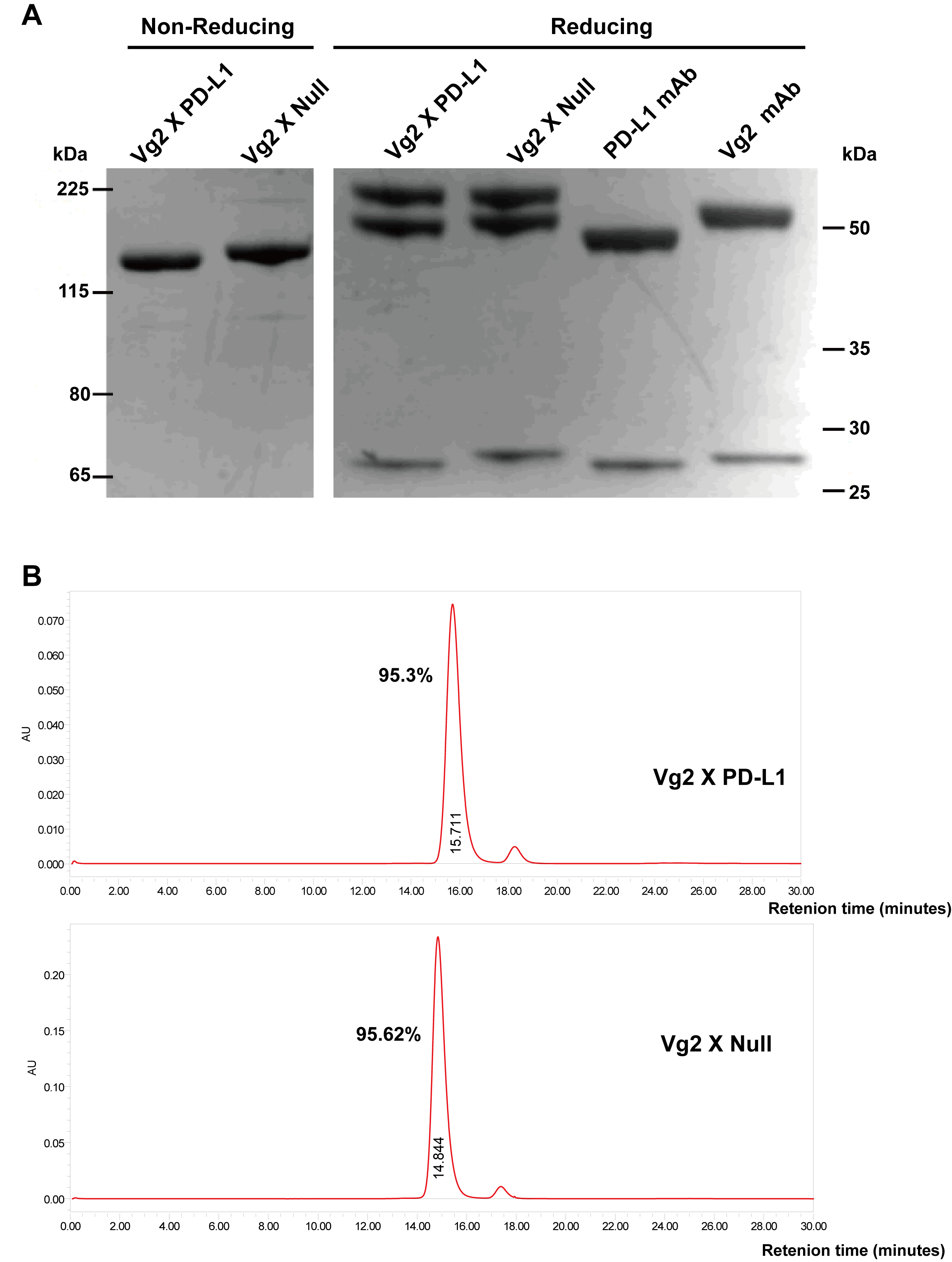

Supplement: Supplementary Figure 1 — Biochemical analysis of generated Vγ2Vδ2 T cell-targeting bsAbs. (A) SDS-PAGE analysis of purified antibodies under non-reducing (left) and reducing (right) conditions. Molecular weight (MW) was indicated in kDa for protein marker. There were 3 and 2 bands for bsAb and mAb, respectively, under reducing conditions as expected. (B) Size exclusion chromatograms of test antibodies (upper, Vγ2 X PD-L1, bottom, Vγ2 X Null). The antibodies were purified by Protein-A and ion-exchange chromatography. The purity of prepared bsAb was more than 95%. [file Image_1.tif]

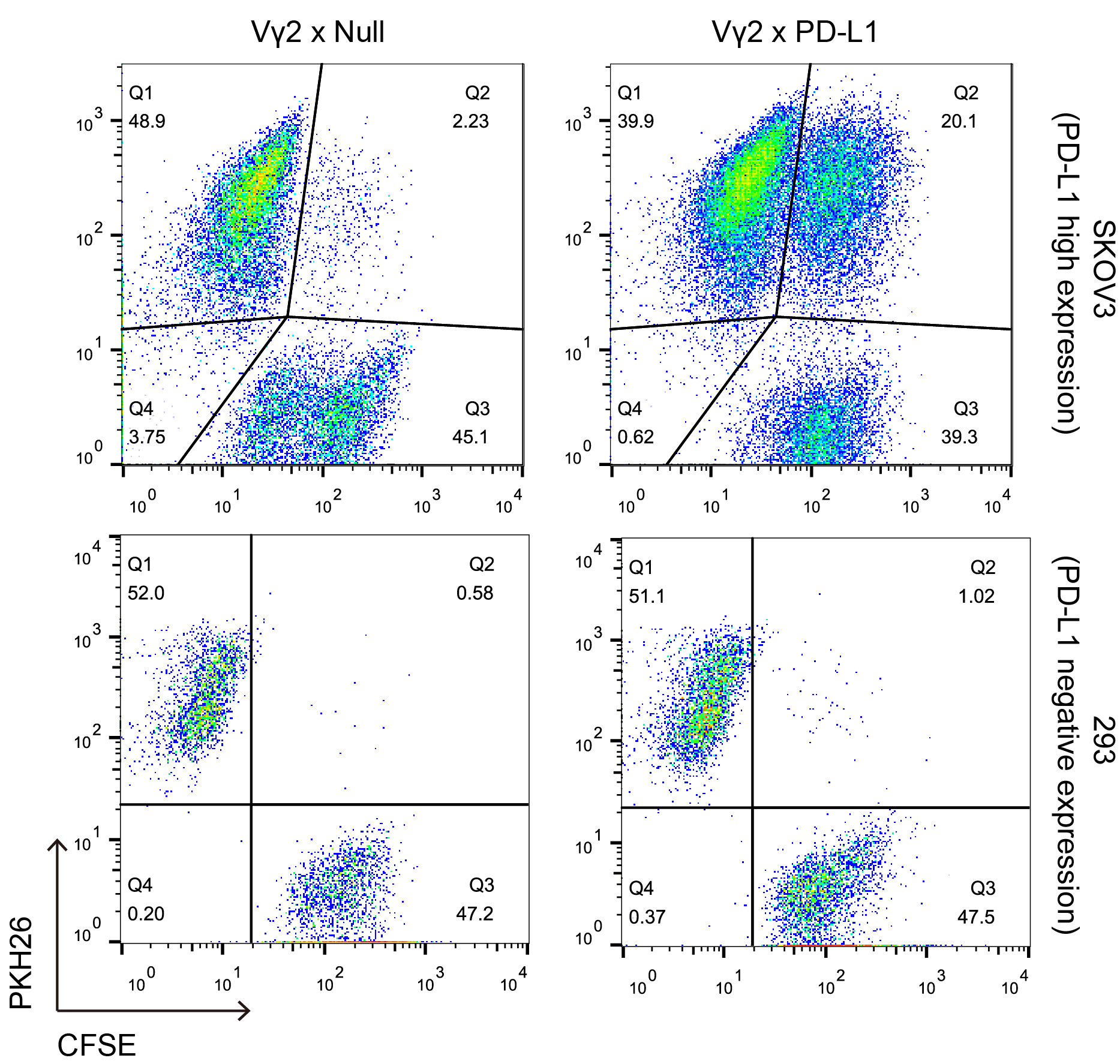

Supplement: Supplementary Figure 3 — Vγ2 x PD-L1 recruited Vγ2Vδ2 T cells to form cell-to-cell conjugates with SKOV3 cells, but not with 293T cells. 0.1 M CFSE-labelled Vγ2Vδ2 T cells were incubated with 0.1 M PKH26-stained SKOV3 cells in the presence of 1 μg/mL (8 nM) Vγ2 x Null (Left) or Vγ2 x PD-L1 (Right) for 30 minutes, then the percentages of CFSE and PKH26 double positive cells (Q2) were depicted as cell-to-cell conjugates. Representative flow cytometric dot plots from three independent experiments (for SKOV3) and three wells (for HEK-293) were shown. Please noted that the upper panel used Vγ2Vδ2 T cells negatively enriched from fresh PBMC cultures treated with Zol+IL2 for 14 days, the bottom panel used Vγ2Vδ2 T cells enriched from cryopreserved PBMC cultures treated with Zol+IL2 for 14 days; and Vγ2Vδ2 T cells were expanded from different donors for SKOV3 and HEK-293. [file Image_3.tif]
